# Chemical and Biocatalytic Synthesis of Capsaicinoids and Capsinoids: An Updated Review

**DOI:** 10.3390/biom16070961

**Published:** 2026-06-29

**Authors:** Guang-Hui Lu, Jian-Rong Dai, Zifu Ni, Zhu Qiao, Lin Zheng

**Affiliations:** 1 School of Biological and Food Engineering, Huanghuai University, 76 Kaiyuan Road, Zhumadian 463000, China; zhenglin@huanghuai.edu.cn; 2 Foshan Shunde Midea Electrical Heating Appliances Manufacturing Co., Ltd., Foshan 528300, China; zhuxiaotang4791@gmail.com; 3 School of Biological Engineering, Henan University of Technology, 100 Lianhua Street, Zhengzhou 450001, China; benzf@haut.edu.cn

**Keywords:** capsaicinoids, capsinoids, biological activity, chemical synthesis, biosynthesis

## Abstract

Capsaicinoids have garnered significant attention due to their diverse bioactivities, including anti-inflammatory, antioxidant, and antitumor effects. However, their strong pungency severely limits their applications in food and pharmaceutical industries. In contrast, capsinoids retain similar biological activities without the irritating pungency, demonstrating much broader application potential. Nevertheless, both classes of compounds naturally occur in low concentrations in plants, rendering large-scale applications challenging. Therefore, the development of efficient and sustainable synthetic methods is urgently needed. Recent advances in novel catalytic technologies have opened new avenues for the green and efficient synthesis of capsaicinoids and capsinoids. This review systematically summarizes the current state of research on the synthesis of these compounds, with a focus on the characteristics of different synthetic methods and their impacts on the resulting bioactivities. Future directions for the development of synthetic approaches are also proposed, aiming to provide a theoretical foundation for further research and application expansion, thereby facilitating the maximal exploitation of their potential value.

## 1. Introduction

**Figure 1:**
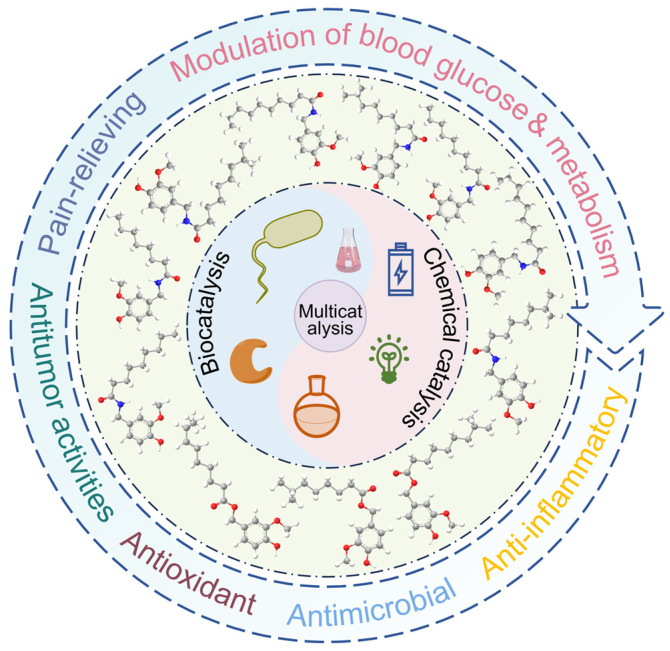
Multicatalytic synthesis of capsaicinoids and capsinoids. Ball-and-stick models of the target molecules (color code: C, grey; H, white; O, red; N, blue).

Native to Central and South America, peppers (*Capsicum* ssp.) have been widely cultivated and consumed across the globe following extensive introduction, domestication, and industrial development [1,2]. Beyond serving as a vital spice that imparts a distinctive pungent flavor to culinary dishes, peppers are abundant in diverse bioactive compounds [3,4]. Recent studies have continuously unveiled the diverse biological properties of these compounds. Beyond their well-known pain-relieving and anti-inflammatory activities, these compounds exhibit antimicrobial, antioxidant, and antitumor activities, as well as the modulation of blood glucose and metabolism (Figure 1) [5,6,7,8,9,10,11,12,13,14,15,16,17,18,19]. These beneficial properties are primarily attributed to its characteristic bioactive constituent—capsaicin (C_18_H_27_O_3_). For example, García-Gasca et al. revealed that capsaicin possesses potent antitumor properties against at least 74 cancer types, primarily through the induction of cytotoxicity and immune adjuvant activity [12]. Elmas and Gezer proposed that capsaicin effectively promotes weight control via multiple mechanisms, such as the regulation of adipocyte lipolysis, increased satiety, elevated energy expenditure, and decreased energy intake [19]. However, the intense pungency of capsaicin has significantly constrained its in-depth development and application within the functional food and pharmaceutical sectors. Japanese researchers first isolated capsinoids (such as capsaicin (C_18_H_27_O_4_) and dihydrocapsaicin) in the non-pungent cultivar (*Capsicum annuum* “CH-19 Sweet”), which was found to accumulate these compounds instead of typical capsaicinoids [20,21,22,23]. Studies have demonstrated that capsiate exhibits biological activities analogous to those of capsaicin but lacks its pronounced pungency, thereby presenting far broader application prospects [22,23].

Given the remarkable bioactivities of capsaicinoids and capsinoids, their medicinal properties have been extensively and intensively investigated by the scientific community, laying a solid theoretical foundation for their large-scale application. However, constrained by their low natural abundance in plants [24,25,26,27,28], the development of efficient and precise chemical or biosynthetic strategies to reduce production costs has emerged as a critical issue demanding urgent resolution. Accordingly, this review systematically summarizes recent advances in the synthesis of capsaicinoids and capsinoids. It critically evaluates the strategic characteristics and reaction efficiencies of various synthetic routes, aiming to provide a theoretical reference for the development of novel synthetic methodologies and their practical applications (Figure 1). Finally, based on the current research landscape, this article outlines future directions in the field, offering scientific insights to facilitate the industrialization of capsaicinoids and capsinoids.

## 2. Synthesis of Capsaicinoids

### 2.1. Chemical Architecture of Capsaicin

**Figure 2:**
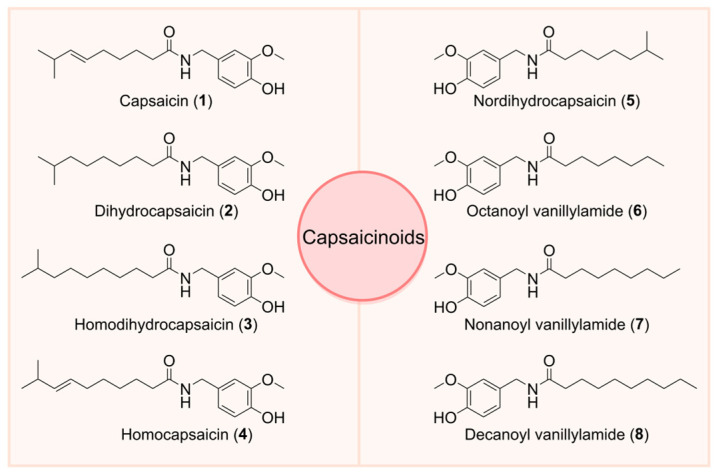
Structures of capsaicinoids.

Capsaicin crystals were first isolated by Thresh in 1876, who formally named the compound “capsaicin” [29]. In 1919, Nelson et al. elucidated the chemical structure of capsaicin [30], identifying it as 8-methyl-*N*-vanillyl-6-nonenamide (Figure 2) [30,31,32,33,34,35,36]. Structurally, capsaicin is an amide compound formed by the condensation of vanillylamine and a branched-chain fatty acid. The unique vanillylamide moiety within its molecule is the key determinant responsible for its intense pungency. Subsequent investigations have revealed that, in addition to capsaicin, peppers contain a variety of structurally related homologs [34,35]. In 1961, Kosuge and Inagaki collectively termed this class of compounds “capsaicinoids” [37]. To date, the identified capsaicinoids primarily include capsaicin (**1**), dihydrocapsaicin (**2**), homodihydrocapsaicin (**3**), homocapsaicin (**4**), nordihydrocapsaicin (**5**), octanoyl vanillylamide (**6**), nonanoyl vanillylamide (**7**), and decanoyl vanillylamide (**8**) [34,35,38]. Studies have demonstrated that **1** and **2** are the predominant pungent components in chili peppers, collectively accounting for over 90% of the total capsaicinoids [16,33,35,39,40,41]. Consequently, extensive research has been dedicated to extracting **1** from peppers utilizing various techniques, including conventional solvent extraction, Soxhlet extraction, supercritical fluid extraction (SFE), microwave- and ultrasound-assisted extraction, as well as green solvent-assisted extraction [7,42,43,44,45,46,47,48,49]. However, the inherently low abundance of capsaicinoids in natural raw materials poses a significant challenge to meeting the demands of large-scale industrial applications. Consequently, academic attention has increasingly shifted toward the exploration of chemical and biosynthetic pathways. In this context, this section will systematically review and summarize recent advances in synthetic strategies from the following two aspects.

### 2.2. Chemical Synthesis

**Figure 3:**
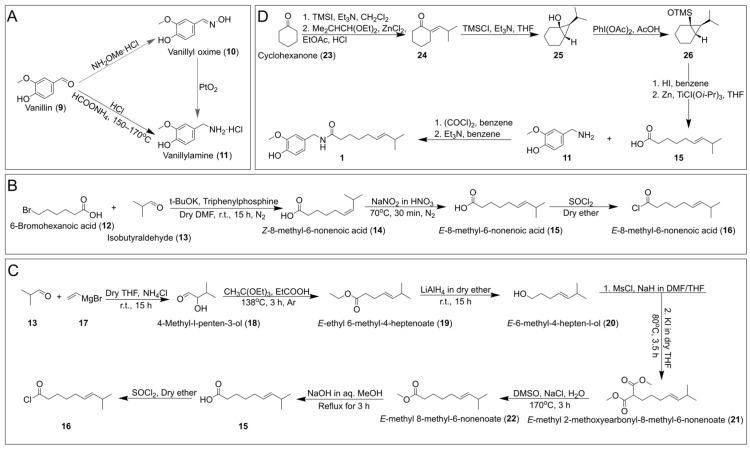
Chemocatalytic synthesis of vanillylamine from vanillin (**A**). Chemical synthesis of *E*-8-methyl-6-nonenoic acid (**B**). Highly stereoselective synthesis of *E*-8-methyl-6-nonenoic acid (**C**). Chemocatalytic synthesis of capsaicin from cyclohexanone and vanillylamine (**D**).

As a key precursor for capsaicinoid synthesis, vanillylamine (**11**) is typically prepared via two routes: the first involves the condensation of methylamine hydrochloride with vanillin (**9**) to yield vanillylidene oxime (**10**), followed by catalytic hydrogenation using platinum(IV) oxide (PtO_2_); the second proceeds through the direct Leuckart reaction of **9** (Figure 3A) [7,50]. Regarding the construction of the other key precursor for **1** synthesis—the branched-chain fatty acid—Kaga et al. first synthesized *Z*-8-methyl-6-nonenoic acid (**14**) via the reaction of 6-bromohexanoic acid with isobutyraldehyde, which yielded the product accompanied by a minor amount of the *E*-isomer (*E*/*Z* = 1:11) [51]. Subsequently, the cis-carbon-carbon double bond (C=C) of this intermediate was catalytically isomerized using nitrous acid (HNO_2_) at 70 °C, successfully shifting the *E*/*Z* ratio to 8:1 and thereby enriching the trans-configured intermediate (**15**). Finally, the resulting **15** was converted into the corresponding acyl chloride (**16**) using thionyl chloride (SOCl_2_) (Figure 3B). To further enhance the stereoselectivity of **15**, Kaga et al. subsequently developed a strategy based on orthoester Claisen rearrangement, which significantly optimized the *E*/*Z* ratio to over 100:1 (Figure 3C) [52]. Meanwhile, Kulinkovich et al. successfully prepared the key intermediate for **1** synthesis, *exo*-7-isopropylbicyclo[4.1.0]heptan-1-ol (**25**), starting from readily available cyclohexanone. Subsequently, this intermediate underwent a ring-opening reaction in an acetic acid medium to yield **15**, which was ultimately converted into **1** (Figure 3D) [53].

**Figure 4:**
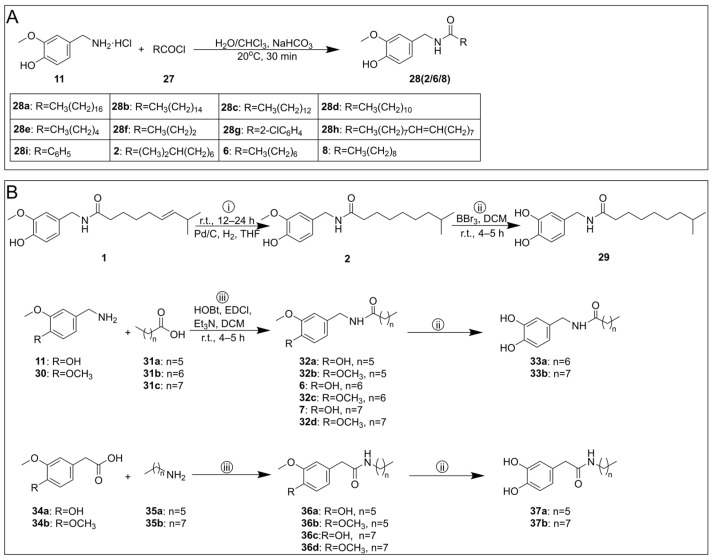
Chemocatalytic synthesis of capsaicinoid compounds from fatty acid acyl chlorides and vanillylamine in a two-phase system (**A**). Chemocatalytic synthesis of capsaicin analogs (**B**).

To overcome the bottlenecks inherent in traditional **1** synthesis routes—specifically the “solvent paradox” arising from the poor solubility of **11** in anhydrous systems coupled with the high susceptibility of acid chlorides to hydrolysis, along with the resulting substantial fluctuations in yield—Tang and Qiu et al. developed a novel H_2_O/CHCl_3_ biphasic reaction system for synthesizing **1** [54]. This system effectively suppressed the hydrolysis of acid chlorides and the bis-acylation side reaction of **11**, significantly boosting the yield of the target product to 93–96%. Furthermore, the reaction system demonstrated excellent substrate generality. A wide range of acid chlorides—including saturated fatty acid chlorides (C4-C18), unsaturated fatty acid chlorides (e.g., oleyl chloride), and aromatic acid chlorides (e.g., benzoyl chloride, *o*-chlorobenzoyl chloride)—underwent highly selective conversion within 30 min, enabling the efficient synthesis of various capsaicinoids (Figure 4A). Yan et al. chemically synthesized a series of capsaicin analogs and systematically evaluated their antioxidant activities and neuroprotective mechanisms [55]. This study designed multiple synthetic routes (Figure 4B): First, using **1** as the starting material, **2** was prepared via hydrogenation (reaction i), or **29** was obtained through a cascade of hydrogenation and demethylation (reaction ii). Second, compounds **32a**–**d**, **6**, and **7** were synthesized via the acylation of 3-methoxybenzylamine derivatives with fatty acids (reaction iii), which were subsequently converted into **33a** and **33b** through demethylation (reaction ii). Finally, acylation (reaction iii) of 3-methoxyphenylacetic acid derivatives with fatty amines yielded compounds **36a**–**d**, which were then subjected to demethylation (reaction ii) to afford **37a** and **37b**. Furthermore, the structure–activity relationships (SARs) of these compounds were elucidated through free radical scavenging assays. Through free radical scavenging assays, the SARs of the aforementioned compounds were elucidated. Antioxidant activity evaluations revealed that **32a** exhibited the most potent antioxidant capacity in the 2,2′-azinobis-(3-ethylbenzthiazoline-6-sulphonate) (ABTS) assay system. Cytotoxicity tests indicated that compound **32a** showed no significant toxicity toward the SH-SY5Y neuroblastoma cell line. Furthermore, measurements of intracellular reactive oxygen species (ROS) and glutathione (GSH) levels further confirmed that **32a** not only effectively alleviates hydrogen peroxide (H_2_O_2_)-induced oxidative stress but also exhibits significant neuroprotective efficacy.

**Figure 5:**
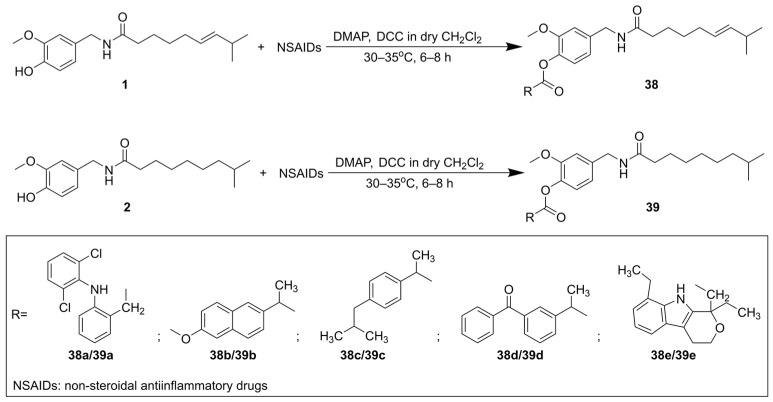
Chemocatalytic esterification of the vanillyl phenolic hydroxyl of capsaicin.

Given that the intense pungency of natural capsaicinoids restricts their broader applications, Zhou and Huang et al. utilized natural **1** and **2** as starting materials to synthesize corresponding derivatives. The vanillyl phenolic hydroxyl was esterified with five non-steroidal anti-inflammatory drugs (NSAIDs) in the presence of *N*,*N*′-dicyclohexylcarbodiimide (DCC) as a dehydrating agent and 4-dimethylaminopyridine (DMAP) as a catalyst, yielding the corresponding capsaicinoid derivatives (Figure 5) [56]. Bioactivity assays demonstrated that, compared to natural **1**, the synthesized derivatives exhibited significantly reduced pungency. Notably, **39d** and **39e** displayed promising anti-inflammatory and analgesic activities, highlighting their potential for development as analgesic agents.

**Figure 6:**
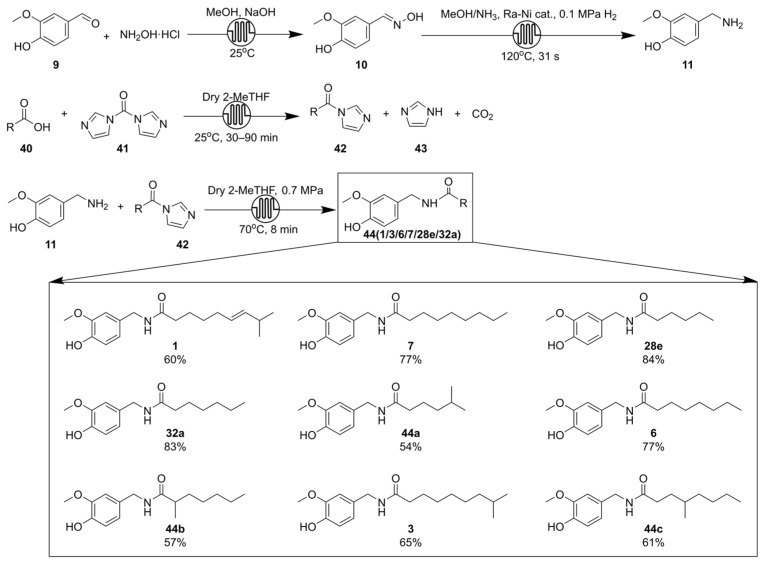
Chemocatalytic synthesis of capsaicin analogs in a semi-continuous-flow system.

To address the bottlenecks inherent in traditional synthetic processes for capsaicinoids—such as prolonged reaction times, fluctuating yields, and substantial generation of chemical waste [55]—Bálint et al. pioneered a semi-continuous-flow reaction strategy, representing a distinct departure from conventional batch synthesis [57]. This protocol specifically comprises three key steps: First, **9** is efficiently converted into **10** in a continuous-flow reactor. Subsequently, this intermediate undergoes continuous-flow catalytic hydrogenation to generate **11**. Finally, **11** is subjected to *N*-acylation with fatty acids activated by carbonyldiimidazole (CDI), ultimately affording the target capsaicinoids. This semi-continuous-flow synthetic system not only enables the highly efficient preparation of **1** but also demonstrates excellent substrate generality, making it widely applicable for the synthesis of various capsaicin analogs with yields ranging from 54% to 84% (Figure 6). Building upon this foundation, Bálint et al. employed cyclodextrins, characterized by a hydrophilic exterior and a hydrophobic interior, to encapsulate the capsaicinoids synthesized via the semi-continuous-flow process [58]. This strategy aimed to improve their water solubility, thermal stability, and bioavailability, while simultaneously masking the undesirable flavors and odors associated with the hydrophobic guest molecules. The results indicated that *β*-cyclodextrin exhibited the most pronounced solubilizing effect on **1** and **2**, whereas *α*-cyclodextrin demonstrated the optimal enhancement of aqueous solubility for **7**. Furthermore, the depth and strength of the inclusion behavior were primarily governed by the structural characteristics of the aliphatic side chains. Specifically, linear and conformationally flexible non-amide side chains displayed the most extensive inclusion modes, while side chains bearing branched or unsaturated moieties resulted in weakened host–guest interactions.

**Figure 7:**
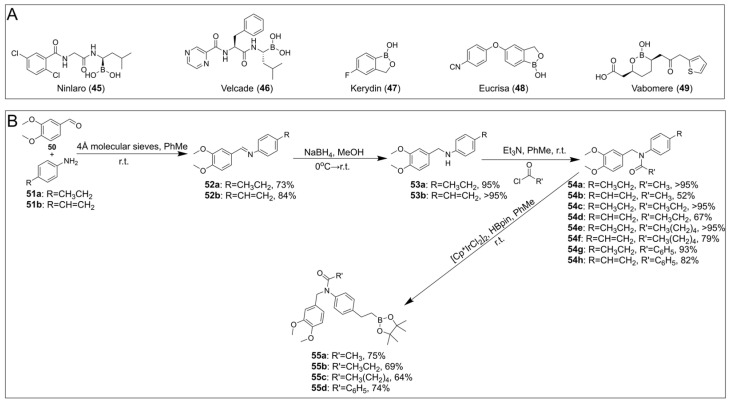
List of typical boron-containing drugs (**A**). Chemocatalytic synthesis of boron-containing capsaicin analogs (**B**).

Given that the incorporation of boron typically significantly enhances the biological activity of natural products—as exemplified by the five boron-containing drugs approved by the U.S. Food and Drug Administration (FDA), including the anticancer agents Ninlaro^®^ [59,60] and Velcade^®^ [61], the antifungal agent Kerydin^®^ [62], the anti-inflammatory agent Eucrisa^®^ [63], and the broad-spectrum antibiotic Vabomere^®^ [64] (Figure 7A)—Rourke and Melanson et al. synthesized a series of boron-containing capsaicin analogs and systematically compared their anticancer activities with those of their non-boron counterparts [65]. Although this synthetic route requires an inert gas atmosphere, prolonged reaction times, and laborious separation and purification of intermediates, the target compounds were successfully prepared via a highly efficient cascade reaction encompassing 3 to 4 steps, with yields ranging from 64% to 95% (Figure 7B). However, cellular anticancer activity evaluations revealed that the non-boron capsaicin analogs exhibited significantly superior activity compared to their boron-containing derivatives. This finding stands in stark contrast to the prevailing conclusion in the literature that “boron incorporation enhances biological activity”. Furthermore, the research team plans to re-evaluate the biological activities of the boron-containing capsaicin analogs synthesized in their previous work [66].

**Figure 8:**
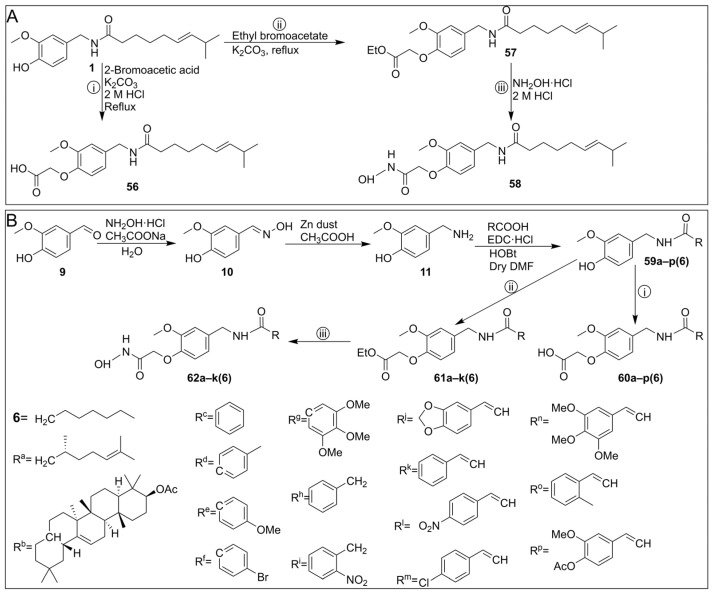
Synthesis of single-point modified capsaicin (**A**). Synthesis of double-modified capsaicin (**B**).

To mitigate the high cytotoxicity of **1** while preserving its biological activity, Shafi et al. performed dual chemical modifications on both the vanillyl phenolic hydroxyl and the hydrophobic long-chain moiety [67]. For instance, novel derivative **56** was synthesized by refluxing **1** in an acetone solution containing 2-bromoacetic acid and potassium carbonate (K_2_CO_3_). Similarly, compound **57** was prepared through the reflux reaction of **1** with ethyl bromoacetate in the presence of K_2_CO_3_, which was subsequently treated with hydroxylamine hydrochloride to yield derivative **58** (Figure 8A). Compound **9** was converted into **11** via a two-step chemical transformation. Subsequently, **11** underwent coupling with various natural or synthetic carboxylic acids mediated by *N*-Ethyl-*N*′-(3-dimethylaminopropyl)carbodiimide hydrochloride (EDC·HCl) and hydroxybenzotriazole (HOBt) to yield singly modified capsaicin analogs **59a**–**p** and **6**, thereby allowing for the modulation of their lipophilic properties. In the presence of K_2_CO_3_, capsaicin analogs **59a**–**p** and **6** were treated with 2-bromoacetic acid under reflux conditions to afford novel acid conjugates **60a**–**p** and **6**, which feature dual-site modifications. Likewise, compounds **59a**–**k** and **6** were treated with ethyl bromoacetate in the presence of K_2_CO_3_ to afford the corresponding esters **61a**–**k** and **6**. Finally, the alcoholic solution of the esters was treated with hydroxylamine to afford the desired products **62a**–**k** and **6** (Figure 8B).

### 2.3. Biocatalytic Synthesis

**Figure 9:**
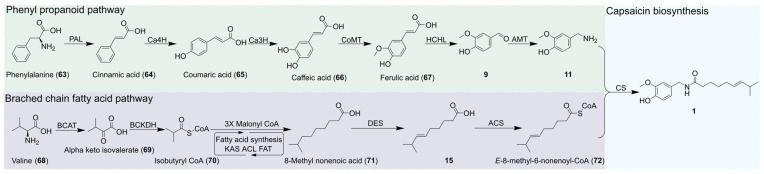
Biosynthetic pathway of capsaicin in chili peppers; PAL, phenylalanine ammonia lyase; Ca4H, cinnamic acid 4-hydroxylase; Ca3H, coumaric acid 3-hydroxylase; CoMT, caffeic acid O-methyltransferase; HCHL, hydroxyl cinnamyl-CoA hydrase/lyase; AMT, aminotransferase; BCAT, branched-chain amino acid transferase; BCKDH, branched-chain α-ketoacid dehydrogenase; KAS, β-ketoacyl-ACP synthase; ACL, acyl carrier protein; FAT, fatty acid thioesterase; DES, desaturase; ACS, acetyl-CoA synthetase; CS, capsaicin synthase.

As illustrated in Figure 9 [4,6,68,69,70,71,72], the natural biosynthetic pathway of **1** in the placenta of *Capsicum* fruits involves the metabolic conversion of phenylalanine to **11** and L-valine to *E*-8-methyl-6-nonenoyl-CoA (**72**), which subsequently undergo condensation catalyzed by capsaicin synthase (CS) to yield **1**. This process entails the coordinated regulation of approximately 18 genes. Among them, PAL1, pAMT, KAS, and PUN1 have been identified as core candidate genes encoding key enzymes, corresponding to phenylalanine ammonia-lyase (PAL), putative aminotransferase (pAMT), ketoacyl-ACP synthase (KAS), and CS, respectively [73,74]. In addition to the positive regulation exerted by MYB [75,76,77,78,79,80] and ERF [81] transcription factors, a recent study demonstrated that the bHLH transcription factor C*c*bHLH68 also acts as a positive regulator of **1** biosynthesis [82]. Specifically, silencing the C*c*bHLH68 gene resulted in a 41% reduction in **1** content, whereas its overexpression led to a 114% increase. Further mechanistic analysis revealed that C*c*bHLH68 exerts its regulatory effect primarily by activating the transcription of C*c*COMT, a key gene in the **1** biosynthetic pathway [83].

Furthermore, environmental factors also significantly influence **1** accumulation in *Capsicum* species [84]. For instance, exposure to blue light has been shown to enhance **1** biosynthesis specifically within the placental tissue of postharvest fruits [85]. This treatment promotes the accumulation of **1** and **2**, concomitant with elevated PAL and branched-chain amino acid transaminase (BCAT) activities and upregulated biosynthetic gene expression. Additionally, water deficit promotes the localized accumulation of these pungent compounds in the placental septum, thereby enhancing the overall spiciness of the fruits [74]. Correspondingly, Shams et al. found that although salt stress inhibited the vegetative growth of *Capsicum* plants, it remarkably promoted the biosynthesis of **1** and **2** [86]. Mechanistic investigations attributed this phenomenon to the salt stress-induced upregulation of PAL1, pAMT, and PUN1 genes in the roots, which subsequently drove the increase in capsaicinoid content. Plant cell culture represents another pivotal strategy for the production of high-value metabolites such as **1** [70,87,88]. This process can be optimized through the modulation of nutrient stress, pH environment, and precursor feeding. Studies have demonstrated that the deprivation of specific nutrients typically induces **1** accumulation, albeit at the expense of biomass growth; conversely, the exogenous addition of biosynthetic precursors or intermediate metabolites can significantly promote **1** biosynthesis. In contrast, Mora-Poblete et al. reported that water stress compromises reproductive growth parameters and fruit pungency while significantly enhancing antioxidant activity in peppers harvested 45 days after flowering [89].

**Figure 10:**
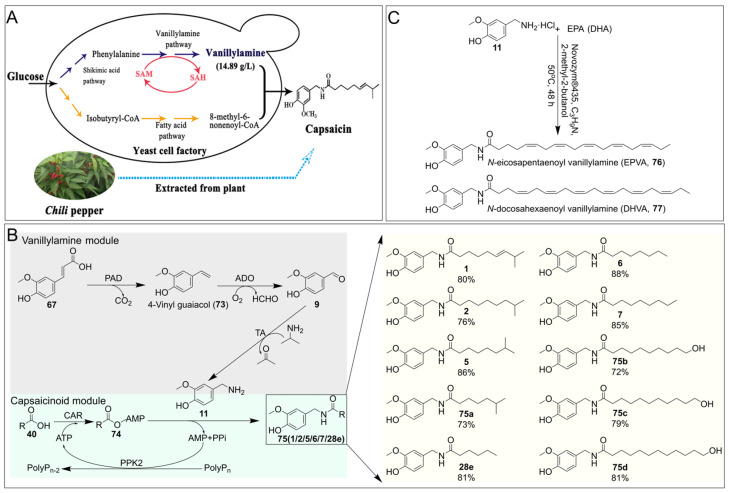
Biosynthesis of capsaicin using a yeast cell factory, reproduced with permission from Ref. [90], Copyright 2023, American Chemical Society (**A**). Total biocatalytic synthesis of capsaicin and its analogs (**B**). Lipase-catalyzed synthesis of capsaicin analogs (**C**). PAD, phenolic acid decarboxylase; ADO, aromatic dioxygenase; TA, transaminase; CAR, carboxylic acid reductase; PPK2, polyphosphate kinase; EPA, eicosapentaenoic acid; DHA, docosahexaenoic acid.

To overcome the environmental unfriendliness associated with traditional chemical synthesis of **1**, Zhang et al. first engineered a **11** biosynthetic pathway in yeast (*Saccharomyces cerevisiae*) [90]. By reconstructing the S-adenosylmethionine (SAM) cycle to significantly enhance methyltransferase efficiency, they achieved a remarkable **11** fermentation titer of 14.89 g/L. Subsequently, through the heterologous reconstruction of fatty acid metabolic pathways in this engineered strain, the efficient de novo biosynthesis of **1** was successfully realized within the yeast host (Figure 10A). To expand the structural diversity of capsaicinoid derivatives, Rodríguez et al. utilized natural **1** as a substrate and employed lipase-catalyzed transesterification with vinyl butyrate and vinyl laurate at the phenolic hydroxyl, successfully preparing the corresponding ester derivatives with yields of 80.6% and 57.5%, respectively [91]. To address the drawbacks of chemical synthesis, such as unsustainable raw materials and environmental unfriendliness, as well as the challenges associated with in vivo metabolic biosynthesis—including low pathway efficiency, poor yields, limited product expandability, and insufficient activity of the key enzyme (CS)—Yun and Heo et al. developed a one-pot, two-step fully biocatalytic system for the synthesis of capsaicinoids from bio-based ferulic acid and fatty acids [92]. This catalytic process is composed of two distinct modules: the vanillylamine module and the capsaicinoid module. In the vanillylamine module, ferulic acid (**67**) is catalyzed by phenolic acid decarboxylase to generate the intermediate 4-vinylguaiacol (**73**), which is subsequently converted into **9** by an aromatic dioxygenase (ADO) and finally transformed into **11** via transaminase (TA) catalysis. In the capsaicinoid module, a phenolic acid decarboxylase (PAD) utilizes fatty acids of varying chain lengths as substrates, synthesizing highly reactive acyl-adenylate intermediates through enzymatic reactions to achieve substrate activation. Subsequently, **11** reacts with these activated intermediates to form an amide bond, yielding the target products. To sustain the catalytic cycle and prevent the inhibition of enzyme activity caused by the accumulation of the byproduct AMP, a polyphosphate kinase (PPK_2_) was introduced into the system to enable the in situ regeneration of ATP. Based on this system, a diverse array of capsaicinoids were successfully synthesized (Figure 10B), achieving conversion rates of 72–88%. This study validates the superiority of biocatalytic systems in expanding product structural diversity.

To investigate the influence of fatty acid chain length on the biological activities of capsaicinoids, Plastina and Loizzo et al. employed eicosapentaenoic acid (EPA) and docosahexaenoic acid (DHA) as acyl donors to undergo amidation with **11**, catalyzed by Novozym^®^435 lipase [93]. The reaction was carried out in a mixed-solvent system consisting of 2-methyl-2-butanol and excess trimethylamine at 50 °C for 48 h (Figure 10C). Following filtration, solvent removal, and purification via silica gel column chromatography, the target products—*N*-eicosapentaenoyl vanillylamine (EPVA) and *N*-docosahexaenoyl vanillylamine (DHVA)—were obtained with purities exceeding 98%. Antioxidant activity assays using the ABTS method revealed that EPVA exhibited superior activity compared to natural **1**, while DHVA demonstrated activity comparable to that of the natural standard. Furthermore, these capsaicin analogs displayed more potent inhibitory effects against *α*-glucosidase than against *α*-amylase, highlighting their potential therapeutic value in the treatment of type 2 diabetes.

**Figure 11:**
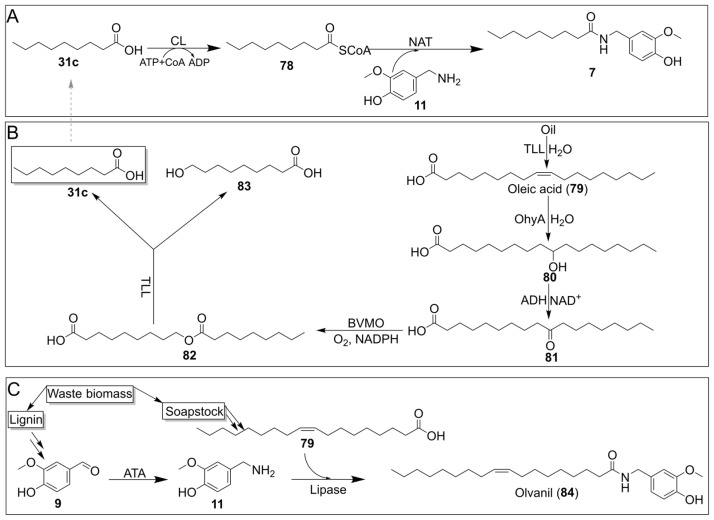
Whole-cell catalyzed synthesis of capsaicinoid (**A**). Whole-cell catalyzed synthesis of capsaicinoid analogs from oil and vanillylamine (**B**). Two-step biocatalytic synthesis of capsaicin analogs from waste biomass (**C**). CL, CoA-ligase; NAT, N-acyltransferase; TLL, *T. lanuginosus* lipase; OhyA, hydratase; ADH, alcohol dehydrogenase; BVMO, Baeyer–Villiger monooxygenase; ATA, aminotransferase; Dashed arrow: **31c** obtained from oils undergoes pathway A to yield **7**.

To develop a synthetic methodology aligned with the principles of green chemistry, Huang and Tessaro achieved the mild and eco-friendly synthesis of capsaicinoids via an all-biocatalytic process utilizing fatty acids derived from natural oils [94,95]. Specifically, Huang et al. engineered *Escherichia coli* to overexpress heterologous CoA-ligase (CL) and *N*-acyltransferase (NAT) genes, facilitating the conversion of **31c** into the active intermediate (**78**), followed by its condensation with **11** to yield **1**, achieving a maximum titer of 0.5 g/L (Figure 11A). More importantly, by introducing a biocatalytic pathway for the conversion of oleic acid to **31c** within this engineered strain, they successfully realized the direct biosynthesis of **1** starting from oil and **11**, reaching a maximum titer of 10.7 mg/L (Figure 11B) [94]. Similarly, Carlquist et al. engineered a yeast strain (*Saccharomyces cerevisiae*) harboring the CL and NAT genes for the whole-cell biosynthesis of **1** from **31c** and **11**, achieving a maximum titer of 10.6 mg/L [96]. Tessaro et al. utilized waste biomass resources, specifically lignin and vegetable oil soapstocks, as sustainable feedstocks to achieve the aminotransferase (ATA)-catalyzed amination of bio-based **9**. The resulting product subsequently underwent lipase-catalyzed amidation with soapstock-derived oleic acid to synthesize **1** (Figure 11C). Collectively, this work established a novel biocatalytic route for **1** synthesis from renewable raw materials [95].

## 3. Synthesis of Capsinoids

### 3.1. Chemical Architecture of Capsinoids

**Figure 12:**
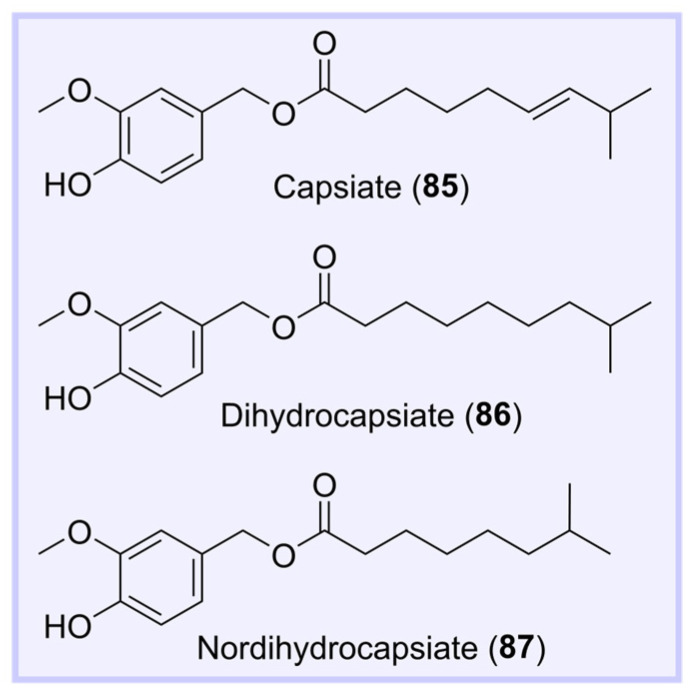
Structures of capsinoids.

Distinct from capsaicinoids characterized by an amide bond, capsinoids are a class of ester compounds formed via the esterification and condensation of vanillyl alcohol (**100**) with fatty acids. Beyond capsiate (**85**), this category also encompasses dihydrocapsiate (**86**), nordihydrocapsiate (**87**), etc. (Figure 12) [9,23,97]. Similarly, although these compounds can be obtained from natural plant matrices using techniques such as conventional solvent extraction, Soxhlet extraction, SFE, and ultrasound-assisted extraction [22,24,26,48,98,99,100,101,102], reliance solely on extraction strategies is insufficient to meet the demands of large-scale applications. This limitation stems primarily from their low abundance in plant substrates and the inherent complexity of downstream separation and purification processes [22]. Consequently, there is an urgent need to develop novel synthetic strategies for their efficient preparation to address escalating market demands and application challenges. Regarding the efficient synthesis of capsinoids, this section will be elaborated from the following two perspectives.

### 3.2. Chemical Synthesis

**Figure 13:**
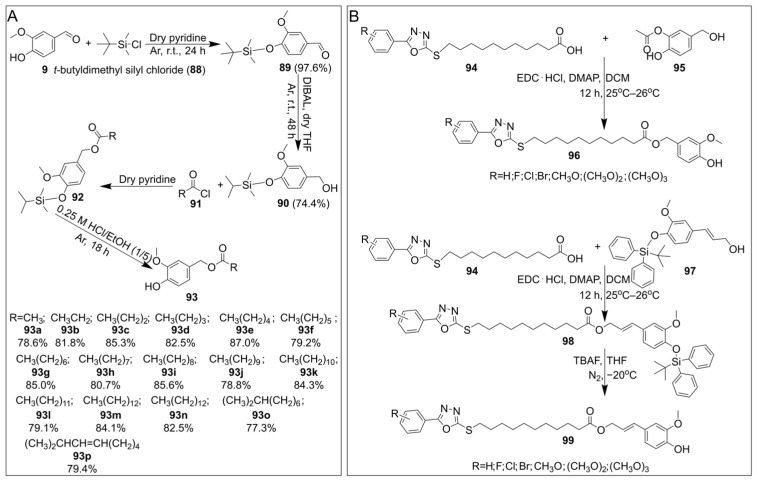
Synthesis of capsinoids from vanillin and fatty acid acyl chlorides (**A**). Synthesis of heterocyclic-containing capsinoid analogs (**B**).

As a versatile strategy, chemical synthesis boasts a long history and widespread application in the de novo synthesis of natural products. It not only enables the efficient preparation of natural compounds but also facilitates the construction of diverse derivatives with varied structures and properties [103,104,105]. To establish a universal synthetic strategy for capsinoids bearing fatty acids of varying chain lengths, Palma et al. developed a four-step protocol comprising phenolic hydroxyl protection, aldehyde reduction, esterification, and phenolic hydroxyl deprotection [106]. Specifically, the phenolic hydroxyl of **9** was first protected via silylation in pyridine. Subsequently, silylated **9** was reduced in tetrahydrofuran (THF) to yield silylated **100**, which then underwent esterification with acyl chlorides in pyridine to generate silylated capsinoids. Finally, a deprotection reaction afforded the target capsinoids (Figure 13A), with yields ranging from 77.3% to 87.0%. Recently, Bonam and Kaki et al. chemically synthesized a series of novel capsinoids, encompassing derivatives bearing heterocyclic fatty acids and vanillyl heterocyclic fatty acids (Figure 13B) [107]. Biological activity evaluations demonstrated that this series of novel capsinoids exhibited significant antioxidant activities, with methoxy substituents further enhancing their antioxidant capacity. Furthermore, these compounds also displayed varying degrees of antibacterial activity.

### 3.3. Enzymatic Synthesis

**Figure 14:**
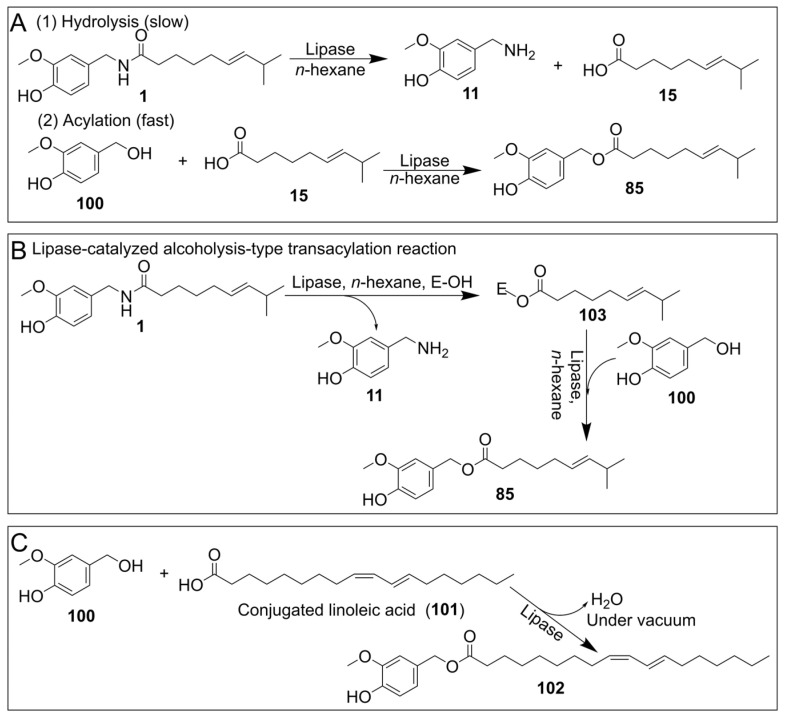
Lipase-catalyzed selective conversion of capsaicin to capsiate (**A**). One-pot lipase-catalyzed synthesis of capsiate from capsaicin (**B**). Lipase-catalyzed synthesis of capsinoids under vacuum (**C**).

Owing to its high efficiency, excellent selectivity, and mild reaction conditions, biosynthesis occupies an indispensable position in the synthesis of natural products and their derivatives [108,109,110]. Numerous studies have systematically investigated the synthesis of capsinoids via transesterification between **100** and fatty acid methyl esters, catalyzed by Novozym^®^435 lipase in organic solvent systems [111,112,113,114]. Ishihara et al. leveraged the kinetic differences between the lipase-catalyzed hydrolysis of natural **1** and the esterification of **100** in an *n*-hexane medium, utilizing immobilized lipase B from *Candida antarctica* to realize the direct transformation of natural **1** into **85** (Figure 14A) [112]. Furthermore, Darszon et al. proposed a one-pot lipase-catalyzed strategy based on solvent engineering for the synthesis of **85** [113]. Employing natural **1** and **100** as substrates in *n*-hexane, this approach utilized Novozym^®^435 to catalyze an alcoholysis-type transacylation, directly converting **1** into **85** without the need for prior hydrolysis (Figure 14B). Furthermore, Kim et al. developed a solvent-free reaction system for the synthesis of capsinoids by catalyzing the esterification of **100** with conjugated linoleic acid using Lipozyme RM IM (a lipase derived from *Rhizomucor miehei*) under vacuum conditions (Figure 14C) [114]. Their research demonstrated that the application of a vacuum significantly enhanced both the reaction rate and product yield. Specifically, under a vacuum ranging from 66.7 kPa to 1.3 kPa, the maximum yield reached 100 mol%, compared to approximately 85 mol% in the absence of a vacuum. Notably, when the vacuum pressure exceeded 13.3 kPa, the maximum yield was achieved within just 3 h. This phenomenon is attributed to the reversible nature of the esterification between **100** and fatty acids, where the generation of water as a byproduct restricts the reaction equilibrium. The vacuum environment effectively removes the generated water, thereby shifting the chemical equilibrium toward the forward reaction and consequently leading to a substantial improvement in both yield and reaction rate.

## 4. Conclusions and Outlook

Capsaicinoids exhibit broad application prospects in the fields of food, medicine, industry, and national defense, owing to their remarkable biological activities, including anti-inflammatory, antioxidant, anti-aging, antitumor, and metabolic regulatory effects. However, their low abundance in natural *Capsicum* species restricts large-scale application, underscoring the urgent need to develop efficient artificial synthetic strategies to compensate for the limitations of natural sources. Furthermore, the intense pungency of capsaicin inherently limits its in-depth research and practical application. In recent years, capsinoids—compounds structurally analogous to capsaicin but devoid of pungency—have garnered increasing attention due to their diverse biological activities. Nevertheless, the extremely low natural abundance of capsinoids similarly necessitates the development of efficient, highly selective, and sustainable synthetic methodologies to facilitate their comprehensive investigation and widespread utilization. Currently, the synthesis of capsaicinoids and capsinoids primarily relies on chemical and biosynthetic approaches. Although these methods have enabled the preparation of the target products, they remain constrained by several limitations, including unsustainable raw materials, non-green reaction processes, the use of toxic solvents, fluctuating yields, limited synthetic routes, low atom economy, and cumbersome post-processing.

The continuous advancement of advanced catalytic and process intensification technologies has ushered in new opportunities for the high-value conversion and synthesis of chemicals, as well as the synthesis of natural products and their analogs. This progress has given rise to a variety of coupled catalytic systems, including photoelectrocatalysis, electrobiocatalysis, photobiocatalysis, and photoelectrobiocatalysis. These synergistic catalytic paradigms not only surmount the bottlenecks associated with traditional thermal catalysis—such as harsh reaction conditions and limited selectivity control—but also enable reactions that are challenging or even unattainable via conventional methodologies. Furthermore, the inherent advantages of mild operating conditions and green sustainability underscore the profound application value of these emerging coupled catalytic technologies.

Therefore, to achieve the highly efficient and selective synthesis of capsaicinoids and capsinoids, future methodological development should focus on the following directions: (1) utilizing renewable and accessible resources (e.g., lignin, vegetable oil) as raw materials to ensure the sustainability of the synthetic process at its source; (2) developing integrated technologies based on synergistic multi-catalytic systems to leverage the complementary advantages of each catalytic system (biocatalysis, chemical catalysis (photo-/electrocatalysis), etc.), thereby avoiding discontinuous, non-green, and overly complex reaction processes; and (3) enhancing the universality and scalability of synthetic methods to accommodate the preparation of diverse structural analogs, thus providing greater potential for expanding their applications. Based on the current research landscape, capsaicinoids and capsinoids continue to hold immense application potential, and breakthroughs in efficient synthetic methodologies will further propel the in-depth exploration of their value. Moreover, the establishment of sustainable synthetic routes not only mitigates the reliance on non-renewable resources, such as fossil fuels, and facilitates the realization of global “dual carbon” goals, but also alleviates the conflict between industrial production and food crop cultivation for arable land.

## Data Availability

No new data were created or analyzed in this study.
